# Effects of Health Qigong Exercise on Lower Limb Motor Function in Parkinson's Disease

**DOI:** 10.3389/fmed.2021.809134

**Published:** 2022-02-17

**Authors:** Xiying Li, Chuanfen Lv, Xiaolei Liu, Xia Qin

**Affiliations:** ^1^School of Physical Education, Yunnan Minzu University, Kunming, China; ^2^School of Physical Education, Zhengzhou University of Light Industry, Zhengzhou, China; ^3^Chinese Traditional Regimen Exercise Intervention Research Center, Beijing Sport University, Beijing, China; ^4^Foreign Language School, Yunnan Minzu University, Kunming, China

**Keywords:** Health Qigong, exercise, Parkinson's disease, lower limbs, motor function

## Abstract

**Purpose:**

This study explored the effects of Health Qigong exercise on lower limb motor function in patients with Parkinson's disease (PD).

**Patients and Methods:**

A total of 40 patients with PD were recruited and randomly allocated into the experimental group and the control group. The experimental group completed an intervention of Health Qigong exercise over 12 weeks, while the control group did not perform any regular physical activity. Data relating to gait, lower-limb joint range of motion, Timed Up and Go, as well as scores for motor function scale from the Unified Parkinson's Comprehensive Rating Scale III (UPCRS III) before and after the intervention were collected for Repeated Measure ANOVA.

**Results:**

Compared to the control group, Health Qigong exercise improved the constant- and high-speed stride length and gait velocity of patients, but not constant- and high-speed stride frequency. Left and right hip flexion and extension range were improved as well as left and right knee flexion range. Finally, Timed Up and Go time became significantly slower and UPCRS III score significantly decreased.

**Conclusion:**

Health Qigong exercise can improve walking ability and lower limb joint range of motion in patients with PD, lessen motor difficulties, and improve the quality of life. This non-pharmacological exercise intervention may be a useful adjustment treatment for PD.

## Introduction

Epidemiologically, there are more than 10 million patients with Parkinson's disease (PD) worldwide, of whom nearly 30% are in China where it represents an annual increase of 100,000 (1). As it develops, PD creates difficulties with walking, destroys the motor function of the lower limbs, impairs the stability and balance of the lower limbs, and further increases the risk of falls. Studies show that about 70% of patients with PD experience falls, leading to secondary harm to patients and long-term disability in severe cases (2).

Studies on lower limb motor function rehabilitation mainly involve the electrical stimulation therapy, mirror therapy, lower limb rehabilitation robots acupuncture, aerobics, square dance, yoga, Tai Chi, and therapy of sports in water (3–10). This study is to apply Health Qigong to explore its effect on lower limb motor function in patients with PD. With the joint evaluation of multiple indicators, the positive effect will be more objective, providing a sound scientific evidence for Health Qigong as a new means of exercise rehabilitation to improve lower limb motor function in patients with PD, which will cut down their economic expense and improve their quality of life. Health Qigong, as a Chinese traditional sport, practices slow and soft tension and tightness in turn, and the body and mind are integrated with harmony (11). Demanding the combination of the center of gravity (CG) with body position and the stability of lower limbs, Health Qigong develops body coordination and bodily mechanisms. It includes Daoyin Health Qigong, Baduanjin Health Qigong, Mawangdui Daoyinshu Health Qigong, Liuzijue Health Qigong, Yijinjing Health Qigong, Wuqinxi Health Qigong, and so on. Health Qigong is becoming increasingly popular because it is simple and easy to learn with low intensity. People can practice Health Qigong anytime and anywhere without any equipment. The unique advantages of Health Qigong have attracted the attention of researchers, with studies confirming that it is valuable for the prevention and treatment of various chronic diseases.

Previous studies have suggested that Health Qigong helps the elderly to improve muscle strength, proprioception, joint range of motion, balance, and body stability of lower limbs. Fu (12) concluded that a 16-week intervention of Baduanjin Qigong increases body flexibility in the elderly, improves the balance of lower limbs, and enhances body stability. Tang (13) proposed that a 3-month intervention of Baduanjin Qigong enlarges the range of motion in the shoulders, neck, elbows, wrists, hips, knees, and ankle joints in elderly women, enhancing body flexibility. Jiang and his team (14) proposed that a 12-week exercise intervention of Health Qigong helps increase the lean mass of the lower limbs in patients with knee osteoarthritis and relieves the pain in the lower limbs. Ma and Zhang (15) found that that a 6-month intervention of Baduanjin Qigong improves balance in the elderly and lessens the risk of falls. Health Qigong helps patients with PD to effectively improve body coordination, stability, muscle rigidity, and gait disorder. Liu et al. (16) proved that, during the practice of Health Qigong, exercising the distal-to-proximal contraction of the lower limbs helps to improve body recognition, strengthens control, and enhances lower limb strength and body stability. Zhang (17) proposed that Wuqinxi Qigong improves gait velocity and balance in PD. The vestibular system and proprioception of patients improve in taking turns and rotations with the left and right limbs. Exercising the muscles of the lower limbs makes them stronger, improving balance, and by imitating the five animals, which is part of the intervention, all the muscles are stretched for a better range of motion in the joints and improved walking ability.

These studies all support the benefits and efficacy of a certain type of Health Qigong separately as an intervention to improve the lower limb motor function of patients with PD. This study was designed to prove whether 12 weeks of the ten exercises combined from the six sets of Health Qigong from Daoyin Health Qigong, Baduanjin Health Qigong, Mawangdui Daoyinshu Health Qigong, Liuzijue Health Qigong, Yijinjing Health Qigong, Wuqinxi Health Qigong can improve the lower limb motor function of patients with PD.

## Methods

### Participants

A total of 40 patients with PD at stages 1–3 as diagnosed by neurologists at Beijing Aerospace Hospital were recruited and randomly allocated into the experimental group and the control group. The sample size was obtained according to the formula: *N* = z2σ2/d2. A written informed consent was obtained from the patients with PD. The inclusion criteria were: (1) meeting the clinical diagnostic criteria for primary PD by the UK Parkinson Disease Association Brain Bank; (2) no changes in medication recently nor during the trial; (3) no deep brain stimulation surgery before or during the study; (4) aged between 50 and 80 years and without cognitive impairment; (5) able to walk without equipment; and (6) not in any other training program for behavioral or pharmacology research. The exclusion criteria were: (1) no other chronic diseases besides PD or neurological damage; (2) poor physical health, impairments to vision, or hearing leading to difficulty in understanding the test; (3) having already learned Health Qigong; and (4) unwilling to provide informed consent to participate. All the participants were required to maintain and record their living activities as usual during the trial, so as to guarantee the experimental data were as true as possible.

### Intervention of Health Qigong

The intervention duration was 12 weeks. The control group did not receive any special exercise intervention during the trial, but after it they could also receive Health Qigong intervention. The participants in the experimental group were instructed to perform the Health Qigong exercise. They attended the exercise five times a week, 60 min each time. Given the clinical motor symptoms and the idea of traditional Chinese medicine that a person keeps healthy as a unity, the intervention involved ten exercises, which practiced the lower limb motor function of patients with PD, combined from the six sets of Health Qigong as promoted by the General Administration of Sport of China. The combined set of Health Qigong exercises includes many lower limb exercises such as moving left or right foot aside, bringing feet together, horse step, heel raising, squat, and standing on a single foot, which drives patients with PD to exercise their lower limbs from multiple angles, dimensions, and ways, so as to have a positive effect on their lower limb motor function. These included Qian Yuan Qi Yun (praying for good fortune at the beginning of the creation of the heaven) and Yun Duan Bai He (white crane flying high in the clouds) in the twelve steps of Daoyin Health Qigong (physical and breathing exercises), Liang Shou Tuo Tian Li San Jiao (holding the hands high with palms up to regulate the internal organs), Wu Lao Qi Shang Wang Hou Qiao (looking backward to prevent sickness and strain), Cuan Quan Nu Mu Zeng Qi Li (thrusting the fist and glaring the eyes to enhance strength) in Baduanjin Health Qigong (eight silken movements), Long Deng (dragon flying) in Mawangdui Daoyinshu Health Qigong, Xu and Xi exercises in Liuzijue Health Qigong (six-character pronunciation), Chu Zhua Liang Chi Shi (showing talons and spreading wings) in Yijinjing Health Qigong, and Niao Fei (flying like a bird) in Wuqinxi Health Qigong (five animals playing). The intervention was led by an experienced Health Qigong instructor who has practiced Health Qigong for more than 3 years, received professional guidance, and won the first prize in the national Health Qigong competition. And the participants followed and performed practice in an exclusive place without outside disturbance.

### Measurement

#### Gait

A Victor Company of Japan (JVC) camera of Canon EOS70D was used for two-dimensional image capture with 50 frames per s. This was placed at the height of 1.4 m from the horizontal ground, a distance of 5 m from the motion plane of patients, and with the principal optical axis perpendicular to the motion plane. A scale of 1 m was used for calibration in all the shooting. The gait test includes the measurement of constant speed and high speed. For the former, the patient walked on flat ground for a distance of 10 m along a straight line at his usual walking pace; for the latter, the patient did the same but at maximum speed. The gait in the middle 3 m was also available for analysis, measured twice at each speed. All gait videos were converted to audio video interleaved (AVI) format by Kinovea software and then the heel points were digitized using visual motion system software to obtain the heel point coordinates for one gait cycle. Each gait cycle was defined as the time from one heel hitting the ground to the same heel hitting the ground again. The horizontal anteroposterior distance between the heels hitting the ground in a gait cycle was stride length, the time for one stride length was gait velocity, and gait velocity divided by stride length was stride frequency. All the gait parameters for constant- and high-speed walking were calculated as the average of the two tests.

#### Lower-Limb Joint Range of Motion

The measuring instrument of the lower-limb motion range was the joint range ruler. For hip flexion, the patient laid supine with the arm fixed by the joint range ruler passing by the trochanter and parallel to the midaxillary line of the trunk. The greater trochanter was the axis and the femur was the longitudinal axis. Afterward, the patient performed knee flexion and leg lift before measurement. For hip extension, the patient laid prone on the same axis, one leg fixed, and mobile leg as described above with fixed pelvis, while it was ensured the patient moved their lower limbs backward as close as possible to the head on the sagittal plane for measurement. For knee flexion, the patient laid supine and then moved the lower leg between the caput fibulae and the lateral malleolus with the lateral femur condylar as the axis and the femur as the longitudinal axis, with the joint range ruler fixed on it. While the patient performed knee flexion and leg lifts, a researcher fixed the pelvis of the patient and held the upper part of the ankle, but did not press it down to make the patient move the heel close to the buttocks for measurement.

#### Timed Up and Go

The instruments for the Timed Up and Go test were a JVC camera of Canon EOS80D and a chair. A chair with a backrest and a height of 45 cm without an armrest was used. In the test area, a measuring tape was used to measure a distance of 3 m in a straight line and mark it. Two-dimensional shootings were taken at 50 frames per s. The camera was placed 1.4 m high from the horizontal ground and 2 m away from the plane of motion of the patient. When the experiment started, patients would stand up, walk 3 m as fast as possible, and then return to the chair again with their back against the backrest to complete the test. Time was recorded on video from when the patient left the backrest of the chair and returned to it. The starting frame was taken as when the patient left the backrest and the ending frame was taken as when the patient returned to the backrest. All videos were named and imported into Kinovea software. The ending frame was subtracted by the starting frame and then was divided by the frame rate to obtain the total time in seconds.

#### Motor Function

Motor function was evaluated using the motor function assessment of the Unified Parkinson's Comprehensive Rating Scale (UPCRS).

### Data Analysis

All the data have been tested for homogeneity test and conformed to normal distribution. SPSS version 23.0 software was used for statistical analysis. The repeated measure ANOVA was used to compare the data relating to lower limb motor function measured before and after the intervention both between and within groups. The Fisher's least square difference method was used for subsequent statistical analysis when the main effect was significant without *time* (pretest and posttest) and *group* (experimental group and control group) interaction. When the interaction effects were significant, a simple effect analysis for differences between the groups and within the group at different time points was conducted. The results are presented as mean ± SD.

## Results

The participants were allocated randomly to either the experimental group or the control group (*n* = 20 in each group). Nine participants dropped out during this study. There were no significant differences in gender, age, height, weight, or disease course between the two groups (*p* > 0.05, Table 1).

**Table 1:** Participant demographics and anthropometrics.

	**Experimental (*n* = 15)**	**Control (*n* = 16)**	***t*-value**	***P*-value**
Gender ratio (male: female)	5:10	6:10		
Age (years)	65.87 ± 6.13	63.25 ± 6.70	1.132	0.597
Height (cm)	162.53 ± 6.90	162.49 ± 6.45	0.016	0.700
Weight (kg)	66.51 ± 7.73	66.84 ± 7.99	−0.117	0.816
Course of disease (years)	5.60 ± 1.72	6.13 ± 1.96	−0.789	0.554

### Gait Parameters Pre- and Posttest by the Group

#### Stride Length

Results indicated that there was a *group* by *time* interaction effect on constant- and high-speed stride length (*p* < 0.05). *Group* and *time* effects were found for high-speed stride length, while only *time* effect was found for constant-speed stride length. The constant- and high-speed stride length of the experimental group significantly increased from pre- to posttest, whereas in the control group it decreased from pre- to posttest but not significantly. There was no significant difference for constant-speed stride length between the two groups either before or after the intervention, while a significant difference after the intervention showed for high-speed stride length. It was implied that over time, Health Qigong exercise had benefits on constant- and high-speed stride length and there was a wider gap between the groups for the latter.

#### Stride Frequency

No significant main and interaction effects were observed for constant- and high-speed stride frequency.

#### Gait Velocity

There was no *group* by *time* interaction effect but a *time* main effect on constant- and high-speed gait velocity (*p* > 0.05). The constant- and high-speed gait velocity of the experimental group significantly increased from pre- to posttest. No differences were found between the groups neither before nor after the intervention. It was shown that over time, Health Qigong exercise benefitted constant- and high-speed gait velocity. All the results of gait outcome measures in pre- and posttest are shown in Table 2.

**Table 2:** Gait outcome measures before and after 12 weeks of intervention.

		**Experimental (*****n*** **= 15)**		**Control (*****n*** **= 16)**		**Time effect**		**Interaction effect**	
		**Pre**	**Post**	**Pre**	**Post**	***f***	***P***	***f***	***P***
Constant-speed	Stride length (m)	0.71 ± 0.11	0.78 ± 0.09^*^	0.74 ± 0.10	0.72 ± 0.09	1.616	0.214	5.516	0.026
	Stride frequency (times/min)	59.72 ± 3.42	61.51 ± 4.68	60.83 ± 4.33	61.35 ± 8.92	1.271	0.269	0.379	0.543
	Gait velocity (m/s)	0.76 ± 0.12	0.90 ± 0.20^*^	0.75 ± 0.15	0.78 ± 0.19	4.380	0.045	1.438	0.240
High-speed	Stride length (m)	0.78 ± 0.09	0.92 ± 0.21^*^	0.80 ± 0.12	0.78 ± 0.98	3.091	0.089	4.219	0.049
	Stride frequency (times/min)	62.30 ± 6.00	63.45 ± 6.21	63.43 ± 6.54	64.51 ± 6.58	1.974	0.171	0.002	0.964
	Gait velocity (m/s)	0.94 ± 0.13	1.07 ± 0.10^*^	0.94 ± 0.12	0.97 ± 0.18	4.344	0.046	1.919	0.177

* *p < 0.05*.

### Lower Limb Joint Range of Motion Pre- and Posttest by the Group

#### Hip Flexion and Extension

Results demonstrated a *group* by *time* interaction effect on left and hip flexion range (*p* < 0.01). A significant *time* effect was found. Left and right hip flexion range in the experimental group significantly increased from pre- to posttest, but no statistical difference was found for the control group pre- to posttest. No differences were observed between the groups either before or after the intervention. It was demonstrated that over time, Health Qigong exercise benefitted left and right hip flexion range of motion.

A *group* by *time* interaction effect was shown in left (*p* < 0.05) and right hip extension range (*p* < 0.01; Table 3). There were significant effects of *group* and *time* in the right, while a *time* effect was there in the left. In the experimental group, the left and right hip extension range significantly increased from pre- to posttest, but no statistical difference was found for the control group pre- to posttest. No differences were found between the groups neither before nor after the intervention for the left but for the right, a significant difference (*p* < 0.01) was thereafter the intervention. It was indicated that over time, Health Qigong exercise benefitted left and right hip extension range of motion and there was a wider gap between the groups for the right.

**Table 3:** Joint range of motion outcome measures before and after 12 weeks of intervention.

			**Experimental (*****n*** **= 15)**		**Control (*****n*** **= 16)**		**Time effect**		**Interaction effect**	
			**Pre**	**Post**	**Pre**	**Post**	***f***	***P***	***f***	***P***
Hip	Flexion	Left	73.40 ± 7.78	79.60 ± 7.50^**^	76.13 ± 8.48	74.44 ± 6.68	3.085	0.090	9.426	0.005
		Right	72.40 ± 10.32	79.07 ± 10.53^**^	76.06 ± 6.48	75.19 ± 11.09	4.794	0.037	8.128	0.008
	Extension	Left	8.20 ± 1.47	10.07 ± 0.88^*^	8.44 ± 1.41	8.06 ± 4.31	1.868	0.182	4.218	0.049
		Right	8.47 ± 1.30	10.27 ± 1.33^**^	8.63 ± 1.02	8.56 ± 0.96	21.629	0.000	24.853	0.000
Knee	Flexion	Left	52.73 ± 9.25	56.80 ± 3.26^*^	54.19 ± 4.48	52.88 ± 3.16	1.245	0.274	4.748	0.038
		Right	54.60 ± 8.75	58.40 ± 3.07^*^	52.81 ± 5.48	50.25 ± 4.78	0.234	0.632	6.198	0.019

* *p < 0.05*;

** *p < 0.01*.

#### Knee Flexion

Results of knee flexion showed a *group* by *time* interaction and significant *group* and *time* effects on left and right knee flexion range (*p* < 0.05). In the experimental group, left and right knee flexion range significantly increased from pre- to posttest, while the decrease from pre- to posttest in the control group was not significant. There was no significant difference between the two groups before the intervention, but a significant difference after it. It was implied that over time, Health Qigong exercise benefitted left and right knee flexion range, enhanced by the wider gap between the groups. All the results of joint range of motion outcome measures in pre- and posttest are given in Table 3.

### Timed Up and Go Pre- and Posttest by the Group

No *group* by *time* interaction effect was observed in the Timed Up and Go test (*p* > 0.05). There was a significant *time* main effect. In the experimental group, the time for Timed Up and Go was significantly shortened from pre- to posttest, while the decrease for the control group was not significant. There were no significant differences between the two groups neither before nor after the intervention. The results showed that over time, Health Qigong exercise benefitted walking ability. All the results of TUG outcome measures in pre- and posttest are shown in Table 4.

**Table 4:** Timed Up and Go outcome measures before and after 12 weeks of intervention.

	**Experimental (*****n*** **= 15)**		**Control (*****n*** **= 16)**		**Time effect**		**Interaction effect**	
	**Pre**	**Post**	**Pre**	**Post**	***f***	***P***	***f***	***P***
Timed Up and Go(s)	11.49 ± 2.26	9.00 ± 2.25^*^	13.78 ± 7.29	13.61 ± 6.57	4.314	0.047	3.255	0.082

* *p < 0.05*.

### Functional Assessment in the Unified Parkinson's Comprehensive Rating Scale III Pre- and Posttest by the Group

Results of the UPCRS III demonstrated a *group* by *time* interaction effect. *Group* and *time* effects were found for motion function (*p* < 0.05). In the experimental group, the score significantly dropped from pre- to posttest, while the score in the control group rose, but not significantly. There was no significant difference between the two groups before the intervention but after the intervention. It was shown that over time, Health Qigong exercise benefitted motor function, enhanced by the wider gap between the groups. All the results of the UPCRS III outcome measures in pre- and posttest are given in Table 5.

**Table 5:** Unified Parkinson's Comprehensive Rating Scale III (functional assessment) before and after 12 weeks of intervention.

	**Experimental (*****n*** **= 15)**		**Control (*****n*** **= 16)**		**Time effect**		**Interaction effect**	
	**Pre**	**Post**	**Pre**	**Post**	***f***	***P***	***f***	***P***
Motor function score (Points)	14.67 ± 1.63	12.47 ± 1.77^**^	14.88 ± 2.42	15.13 ± 3.81	6.497	0.016	10.256	0.003

** *p < 0.01*.

## Discussion

This study aims to explore the effects of Health Qigong exercise on lower limb motor function in patients with PD. The results of this study demonstrate that the intervention involved ten exercises from the six sets of Health Qigong do have positive effects on gait, lower limb joint range of motion, Timed Up and Go test, and the score for motion function in the UPCRS III. Correspondingly, they eventually improve the lower limb motor function in PD. More detailed information will be discussed as follows.

### Gait

Patients with PD suffer from disordered gait and a smaller range of motion in the lower limb joints and their walking ability becomes weak with an unstable center of gravity. Patients with PD fall frequently and may suffer from secondary damage (18). Disordered gait is one of the primary clinical motor symptoms in patients with PD and is the critical cause of lower limb motor dysfunction. As the effects of PD on the central nervous system progress, its regulation of the limb motor system weakens or is even lost (19). The ability of patients with PD to perform daily activities, such as wearing shoes, walking upstairs, or crossing the street alone, decreases and their body control reduces. The muscle strength of the affected side weakens due to a sustained lack of active exercise, which further reduces the proprioceptive input to the joint, restricts the exercise capacity of the patient and reduces balance and stability. The proprioception is weakened. Qigong exercise is one type of mind-body exercise. It involves the cooperation and coordination of all the parts of the body and the mind. While the patient completes a movement, he/she controls the change in the body position, such as moving left or right, lifting the heel, turning the body or the head, and so on. After practicing these complex, slow and changeable exercises, body proprioception significantly improved.

In this study, gait involves stride length, frequency and gait velocity at a constant speed and high speed. After 12 weeks of Health Qigong intervention, the constant- and high-speed stride length of patients and gait velocity significantly increased (although constant- and high-speed stride frequency did not increase). This positive effect was increased as intervention duration was prolonged, especially for high-speed stride length and gait velocity. In the Cuan Quan Nu Mu Zeng Qi Li (thrusting the fist and glaring the eyes to enhance strength) move, the patient squats the legs in a horse stance with the toes fully on the ground, effectively making them concentrate CG on the toes. Movements such as thrusting the fist, grasping, and rotating the wrist for both hands stimulate the meridians of the hands and feet, increasing strength, and making the lower limb muscles more powerful. In the Long Deng (dragon flying) move, the individual squats to stand up slowly then lifts the heels, stretches the legs, and squats down again. The up and down movement of the CG in this process strengthens body stability. Exercising the lower limb muscles, extensibility of ligaments, and the motion range of the hip and knee, increased flexibility of the hip and knee joints, stability of lower limbs, balance, and greater capacity for the exercise of the lower limbs is achieved. In addition, in the Qian Yuan Qi Yun (praying good fortune at the beginning of the creation of heaven) move, Xu exercise move, and Wu Lao Qi Shang Wang Hou Qiao (looking backward to prevent sickness and strain) move, the body and the limbs turn or rotate creating more flexible joints. The nerves, muscles, and joints are stimulated and the proprioception of the limbs and trunk is effectively enhanced. So is core strength. All these lead to better body stability, so that patients with PD enjoyed better walking and suffered less gait disorder.

### Lower Limb Joint Range of Motion

After 12 weeks of Health Qigong exercise, the ranges of the left and right hip flexions and extensions and of left and right knee flexions in patients became significantly broader in the experimental group. The positive effect was enhanced as the intervention duration was prolonged. Besides, the experimental and control groups showed significant differences in the ranges of the right hip extension, left, and right knee flexions.

Patients with PD often experience stiffness and weakness of the lower limb muscles, which is the primary cause of the small motion range of lower limb joints. In the Health Qigong intervention, most strength training of the lower limbs is stance training (and in particular, horse stance training) which is static, such as in the Cuan Quan Nu Mu Zeng Qi Li (thrusting the fist and glaring the eyes to enhance strength) move. Static strength training can more effectively improve muscle tone and enhance neuromuscular control, increasing the stability of the joints. Moreover, changes of walking patterns help to train the quadriceps femoris and hamstrings of the lower limbs, thereby improving muscle strength and increasing the stability of the joints. At the same time, right and left CG movement can also enhance the patient's sense of direction, such as in the Qian Yuan Qi Yun (praying for good fortune at the beginning of the creation of heaven) and Liang Shou Tuo Tian Li San Jiao (holding the hands high with palms up to regulate the internal organs) moves with the change of walking patterns and the shift of the CG between the left and right (20).

In addition, in the Niao Fei (flying like a bird) move, the practitioner is required to complete knee-lifting movements independently on the left or right leg (21) and remain stable during the process. Doing these helps exercise the range of motion of the hip joint, the strength of the legs, and the body stability of the patient. Long-term and regular Health Qigong exercise can effectively enhance lower limb strength and as well as the range of motion, thereby improving the balance and quality of life of patients. In the Yun Duan Bai He (white crane flying high in the clouds) move and Long Deng (dragon flying) move, the heel-lift move can effectively improve the extensibility and strength of the lower limb muscles, and increase ankle joint stability. Imagining themselves as birds or cranes during practice (22), patients keep their lower limbs stable and upper body relaxed. This can effectively train the control ability of all skeletal muscles, strengthening control of the surrounding muscles of the hips, knees, and ankle joints, and improving gait so that the patients with PD walk with stability and flexibility.

### Timed Up and Go Test

For patients with PD, the difficulty to combine standing up and sitting directly reduces their walking ability for muscle weakness, stiffness, and poor balance caused by PD. These are the critical causes of reduced ability to complete daily activities such as sitting down and standing up.

We observed from the results that patients required significantly less time to complete the Timed Up and Go test than they did before the 12 weeks of Health Qigong intervention. The positive impact became increasingly stronger as intervention duration was prolonged. The Yun Duan Bai He (white crane flying high in the clouds) move involves bending the knees to squat down and get up, and the Niao Fei (flying like a bird) move involves squatting down and getting up while kicking a leg. The Cuan Quan Nu Mu Zeng Qi Li (thrusting the fist and glaring the eyes to enhance strength) move requires the practitioner to squat half in a horse-step state and the Long Deng (Dragon Flying) move requires squatting down entirely from being upright and then getting back up. Practicing these moves, the patients will have muscle control and lower limb strength, suffer less rigidity, and enjoy more balanced walking.

### Score for Motion Function in the UPCRS III

As a criterion to evaluate motor function of patients with PD, the UPCRS is composed of four parts. We only applied the third part (motor function rating) to assess lower limb motor function in patients with PD. There are a total of 14 items, each scored 0–4 points. The higher the total score, the more severe the symptoms, and this measure can objectively reflect improvement in motor symptoms in patients with PD.

We found that the scores of motion function of patients significantly decreased after the intervention and the positive effect became stronger as the intervention time lasts longer. In the practice of the Xu and Xi exercises, patients move their lips backward to trigger sounds, which stimulate the facial muscles to improve control of them and help patients with pronunciation. In addition, during practice, teachers and teaching assistants often communicate with patients to ensure the effective completion of movements, while unconsciously encouraging patients to talk to others and effectively train their language skills. In such a positive atmosphere, everyone cares, helps, encourages, and fights against PD. Studies demonstrate that satisfaction in interpersonal relationships helps to enhance the positive mental health of people (23). Health Qigong is an activity that emphasizes harmony between oneself and the outside world, rather than comparing oneself or competing with others. Through this non-competitiveness, Health Qigong has a positive effect on individual personalities and interpersonal skills as well as on mood regulation in a comfortable atmosphere.

In daily life, patients with PD have shaking hands, which affects eating and drinking. When patients with PD are nervous, they have more difficulty in moving their hands freely, which can lead to low self-esteem. Exercise improves fitness, which has a positive correlation with self-efficacy and self-esteem (24). Highly demanding motions in sports promote body perception, which further stimulates the pursuit of health of people. This process can promote both the physical and mental health. The whole set of exercises includes coordinated movements as arm rotation, shoulder extension, wrist folding, finger curling, finger flicking, wrist shaking, flash palm, palm turning, palm supporting, five-finger external supporting, loosening finger, loosening wrist, gripping, and so on. These train the hands, stimulate nerve endings, strengthen nerve conduction, relieve muscle rigidity, and improve shaking hands for better hand function. When patients feel positive change, they are more willing to participate in exercise and this forms a virtuous cycle.

In the whole set of exercises, there are left and right CG movements and up and down movements, such as pushing up, turning the waist left and right, stepping left and right, bringing the feet together, squatting in the horse stance, heel raising, squatting down with knees bent, and standing on one foot with a knee lift. These exercises stretch all the muscles of the whole body, enhance elasticity, increase the range of motion of joints, strengthen the lower limbs, and train body stability and also improve muscle rigidity, postural instability, gait, and reduce the risks of falls.

### Strength and Limitations

This study possesses several strengths: (1) determined eligibility criteria of participants through experiment; (2) similar demographic and anthropometric information (age, gender, course of disease, medication); (3) an independent practice place (no outside interference); (4) randomized controlled experimental design; (5) confounding factors controlled with repeated measure ANOVA; (6) side effects on experimental data effectively avoided without other drug therapy; (7) authenticity, scientific nature and timeliness of the data guaranteed thanks to timely testing right before and after patients' practice for. Meanwhile, some limitations of this study need to be acknowledged. First, this study covered only 12 weeks of Health Qigong intervention, which was relatively short. Second, different data and effects may be generated when patients with PD were involved in lower limb motor function intervention at different stages and if the patients with PD had other simultaneous diseases. Third, the sample size of this study was small for patient dropout, since those patients suffered severe disease or had other personal reasons at the beginning of this study. Fourth, Health Qigong may not be practiced with the same quality and standard degree among the participants. Fifth, the follow-up effects of Health Qigong intervention were not known without subsequent visits for evaluation.

## Conclusion

Health Qigong exercise is a type of mind-body exercise. It involves the cooperation and coordination of all parts of the body and the mind. Patients need to have a high concentration in action during practice, otherwise, stagnation will occur. This exercise stimulates the ability of the brain to control the body with body movements. After 12 weeks of Health Qigong exercise, the gait and lower limb joint range of motion in patients with PD were improved alongside lower limb motor function. The Timed Up and Go test time was reduced, while the walking ability and exercise capacity improved. These benefits also help to improve the quality of life for patients with PD. This study shows that Health Qigong can be used as an auxiliary therapy for exercise rehabilitation in addition to treatments such as drugs and surgery, offering some new ideas for rehabilitation training, which will be of interest to both the researchers and clinical practitioners. From the perspective of clinical benefits, it effectively reduces the social and patients' economic pressure. With its obvious advantages of moderate intensity and being easy to learn, it is suitable for patients with PD. As it is culturally closely related to Chinese people, it has better exercise and treatment compliance.

## Data Availability Statement

The original contributions presented in the study are included in the article/supplementary material, further inquiries can be directed to the corresponding author/s.

## Ethics Statement

The studies involving human participants were reviewed and approved by the Ethics Committee of Beijing Sport University (protocol code 2021002H and January 1st, 2021). The patients/participants provided their written informed consent to participate in this study.

## Author Contributions

XLi, CL, and XLiu contributed in conceptualization. XLi and XLiu contributed in methodology. XLi and CL contributed in formal analysis, writing—original draft preparation, and data curation. CL, XQ, and XLiu contributed in investigation. XQ and XLiu contributed in resources. XLi, XQ, and XLiu contributed in writing—review and editing. XLiu contributed in funding acquisition. All the authors have read and agreed to the published version of the manuscript.

## Funding

This study was funded by the Beijing Sport University International Cooperation Topics (Project number 2020046).

## Conflict of Interest

The authors declare that the research was conducted in the absence of any commercial or financial relationships that could be construed as a potential conflict of interest.

## Publisher's Note

All claims expressed in this article are solely those of the authors and do not necessarily represent those of their affiliated organizations, or those of the publisher, the editors and the reviewers. Any product that may be evaluated in this article, or claim that may be made by its manufacturer, is not guaranteed or endorsed by the publisher.
